# Doxycycline-Loaded Calcium Phosphate Nanoparticles with a Pectin Coat Can Ameliorate Lipopolysaccharide-Induced Neuroinflammation Via Enhancing AMPK

**DOI:** 10.1007/s11481-024-10099-w

**Published:** 2024-01-18

**Authors:** Suzan Awad AbdelGhany Morsy, Mona Hassan Fathelbab, Norhan S. El-Sayed, Salma E. El-Habashy, Rania G. Aly, Sahar A. Harby

**Affiliations:** 1 Clinical Pharmacology Department, Faculty of Medicine, Alexandria University, Alexandria, Egypt; 2 Medical Biochemistry Department, Faculty of Medicine, Alexandria University, Alexandria, Egypt; 3 Medical Physiology Department, Faculty of Medicine, Alexandria University, Alexandria, Egypt; 4 Department of Pharmaceutics, Faculty of Pharmacy, Alexandria University, Alexandria, Egypt; 5 Pathology Department, Faculty of Medicine, Alexandria University, Alexandria, Egypt

**Keywords:** Neuroinflammation, AMPK, TLR-4, Nrf2, IL-6, Doxycycline

## Abstract

Neuroinflammation occurs in response to different injurious triggers to limit their hazardous effects. However, failure to stop this process can end in multiple neurological diseases. Doxycycline (DX) is a tetracycline, with potential antioxidant and anti-inflammatory properties. The current study tested the effects of free DX, DX-loaded calcium phosphate (DX@CaP), and pectin-coated DX@CaP (Pec/DX@CaP) nanoparticles on the lipopolysaccharide (LPS)-induced neuroinflammation in mice and to identify the role of adenosine monophosphate-activated protein kinase (AMPK) in this effect. The present study was conducted on 48 mice, divided into 6 groups, eight mice each. Group 1 (normal control), Group 2 (blank nanoparticles-treated), Group 3 (LPS (untreated)), Groups 4, 5, and 6 received LPS, then Group 4 received free DX, Group 5 received DX-loaded calcium phosphate nanoparticles (DX@CaP), and Group 6 received DX-loaded calcium phosphate nanoparticles with a pectin coat (Pec/DX@CaP). At the end of the experimentation period, behavioral tests were carried out. Then, mice were sacrificed, and brain tissue was extracted and used for histological examination, and assessment of interleukin-6 positive cells in different brain areas, in addition to biochemical measurement of SOD activity, TLR-4, AMPK and Nrf2. LPS can induce prominent neuroinflammation. Treatment with (Pec/DX@CaP) can reverse most behavioral, histopathological, and biochemical changes caused by LPS. The findings of the current study suggest that (Pec/DX@CaP) exerts a significant reverse of LPS-induced neuroinflammation by enhancing SOD activity, AMPK, and Nrf2 expression, in addition to suppression of TLR-4.

**Graphical Abstract:**

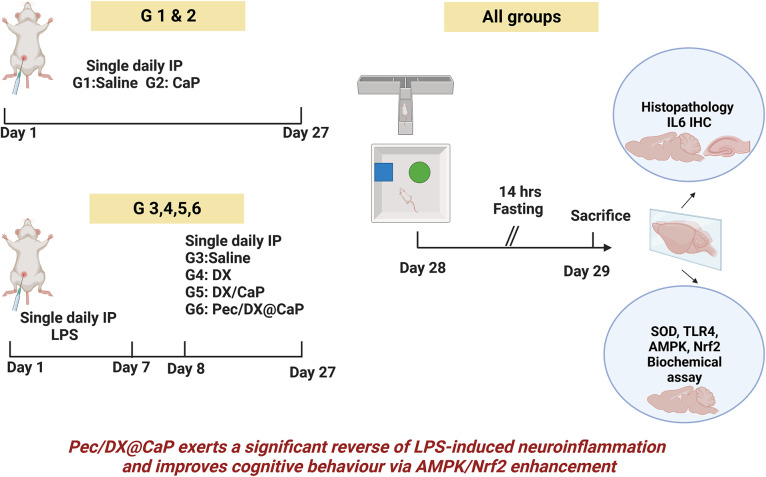

**Supplementary Information:**

The online version contains supplementary material available at 10.1007/s11481-024-10099-w.

## Introduction

Neuroinflammation is a complex inflammatory response that occurs within the central nervous system (CNS) because of multiple triggers like ischemia, infections, trauma, immune reaction, or exposure to toxic proteins. These triggers activate microglia, through stimulation of Toll-like receptor-4 (TLR-4), to secrete neurotrophic factors, reactive oxygen species (ROS), glutamate, chemokines, and cytokines, which initiate a transient inflammatory response (Jurcau and Simion 2021; Takeda et al. 2021). Superoxide is another source of ROS, it produces hydrogen peroxide, hydroxyl radicals, and peroxy-nitrite, which enhance angiogenesis causing disruption of the blood-brain barrier, in addition to promoting protein, lipids, and DNA oxidation ending with tissue damage (Takeda et al. 2021).

On the other hand, defense anti-inflammatory mechanisms are activated in a trial to limit this damaging effect which can occur as a result of over-expressed pro-inflammation. These mechanisms include activation of the endogenous antioxidant enzyme, Superoxide dismutase (SOD), which inhibits the ROS-mediated cell injury (Younus 2018). Another enzyme involved in the control of neuroinflammation is adenosine monophosphate-activated protein kinase (AMPK), it is a heterotrimer enzyme expressed in mammalian cells and exerts antioxidant and anti-inflammatory actions that can be mediated through enhancement of nuclear factor erythroid 2-related factor 2 (Nrf2) activation and suppression of proinflammatory cytokines (Yu et al. 2020). Furthermore it has the ability to convert the microglia from pro- to anti-inflammatory phenotype and suppress autophagy and cell death (Saito et al. 2019).

The prognosis of neuroinflammation and its fate depends on the balance between different mediators favoring or antagonizing the inflammatory processes (Kangralkar et al. 2010). Failure of the endogenous protective mechanisms to stop the tissue damage mediated by these triggering factors, results in persistent neuroinflammation, which can be involved in the pathophysiology of multiple diseases like depression, Alzheimer’s disease, parkinsonism and dementia. In Alzheimer’s disease, neuroinflammation mediates the upregulation of the activated microglia (the pro-inflammatory M1 phenotype) with increased release of the pro-inflammatory factors, mainly interleukin-6 (IL-6). It has been demonstrated that IL-6 is a good biomarker that corresponds with the severity of cognitive impairment. Furthermore, it inhibits the phagocytosis of extracellular β-amyloid plaques in Alzheimer’s disease model mice’s brains (Stamouli and Politis 2016). The activated microglia is present in different brain regions including the cerebral grey matter and hippocampus which shows the highest degree of neurodegeneration, damage, and other pathological changes. Hippocampal atrophy and parahippocampal cortical thinning were related to worse cognition (Rosi et al. 2005; Nicastro et al. 2020). In multiple sclerosis cerebral white matter and hippocampal neuroinflammation with the associated microglial activation participates not only in the neuronal damage and demyelination, but also in the associated depressive symptoms (Colasanti et al. 2016; Ghorbani and Yong 2021).

In the cerebellum, neuroinflammation is involved in the occurrence of ataxia, tremors in addition to disrupted behavior associated with numerous diseases (Parvez and Ohtsuki 2022). Thus, targeting neuroinflammation can be a promising method in the management of these diseases (Dong et al. 2018; Ren et al. 2018).

Doxycycline is a second-generation tetracycline with fewer adverse effects and a higher potential to cross the blood-brain barrier (BBB) in comparison to tetracycline. Recently, it has been repurposed for its antioxidant, anti-inflammatory, anti-amyloidogenic, and anti-apoptotic effects (Balducci et al. 2018; Santa-Cecília et al. 2019). Altoé et al. studied the anti-inflammatory and antioxidant effects of doxycycline on the skin and the impact of these activities on wound healing and concluded that it can increase the level of antioxidant enzymes, namely, SOD and catalase, and improve wound healing. Other studies showed the promising activity of doxycycline against traumatic brain injury. However, it has been reported to possess a dose-dependent cytotoxicity, that is greatly ameliorated by incorporation in composite nanoparticles (Altoé et al. 2021; El-Habashy et al. 2021c).

A recent approach to enhance the physicochemical properties and pharmacological efficacy of drugs used for the treatment of neurodegenerative diseases is nano-drug delivery, using various nanocarriers like inorganic and polymeric nanoparticles, including bioactive polymers. The controlled drug release from nano-drug delivery systems improves drug delivery and effectiveness, prolongs the duration of action and lowers cytotoxicity, while nanocarrier surface modification can boost its therapeutic efficacy (Rahmani et al. 2022). Among bioactive polymers, pectin has recently emerged as a natural, biocompatible polymer with antioxidant and anti-inflammatory properties (Ávila et al. 2021). In this regard, loading doxycycline in a biocompatible delivery system integrating pectin as bioactive entity might offer higher therapeutic potential. Indeed, doxycycline-encapsulated solid lipid nanoparticles showed a higher ability to suppress colonies of intracellular infection, in comparison to free doxycycline (Hosseini et al. 2019).

The current study aimed to assess the potential role of pectin-coated, doxycycline-loaded calcium phosphate nanoparticles (Pec/DX@CaP) in the treatment of lipopolysaccharide (LPS)-induced neuroinflammation in mice and to identify the role of interleukin-6 (IL-6), SOD, TLR-4, AMPK and Nrf2.

## Materials and Methods

### Preparation of Calcium Phosphate Nanoparticles (CaP)

Doxycycline hydrochloride (DX) was received from the European Egyptian Pharmaceutical Industries, Egypt as a gift. Calcium chloride (CaCl2) and disodium hydrogen orthophosphate anhydrous (Na2HPO4) were purchased from Loba Chemie, India and Winlab, England, respectively. Citrus pectin (Classic CU 701 with 37% degree of esterification) was a kind gift from Herbstreith & Fox GmbH & Co. KG Pektin-Fabriken, Germany.

CaP was prepared via wet chemical precipitation as previously reported (El-Habashy et al. 2021), with slight modifications. Briefly, precursor solutions of CaCl2 (0.06 M) and Na2HPO4 (0.035 M) were prepared in deionized water and their pH was adjusted to 11 ± 0.5. Na2HPO4 solution was then dropwise added to CaCl2 solution at 25 °C while stirring (IKA Eurostar; IKA Labortechnik, Germany). The developed dispersion was allowed to age for 1 h, then the CaP pellet was separated by centrifugation (Laboratory centrifuge 3 K-30; Sigma, Germany) at 10,000 rpm and 20 °C for 10 min. The separated pellet was finally washed with ethanol before further use.

### Preparation of DX-Loaded Calcium Phosphate Nanoparticles (DX@CaP)

DX loading was conducted as previously reported (El-Habashy et al. 2021). The separated CaP pellet was dispersed in DX solution (6 mg/mL in ethanol) at DX: CaP w/w ratio of 2:1 and allowed to stir for 24 h. The mixture was then centrifuged at 15,000 rpm and 4 °C for 15 min to obtain the DX@CaP pellet.

### Preparation of Pectin-Coated, DX-Loaded Calcium Phosphate Nanoparticles (Pec/DX@CaP)

Pectin coating was carried out as previously reported (Shehata et al. 2022), with modifications. The separated DX@CaP pellet was first dispersed in 5 mL of deionized water. The formed dispersion was then used to titrate the pectin aqueous solution at different concentrations (0.5 and 1 mg/mL) at a 1:1 v/v ratio. The mixture was further stirred for 30 min before use. Nanoparticle dispersions were either used as prepared or lyophilized (Lyoquest; Telstar, Spain).

### Characterization of the Developed Nanoparticles

#### Measurement of Zeta Potential

The zeta potential of the prepared nanoparticle dispersions was investigated using dynamic light scattering (Malvern Zetasizer NanoZS; Malvern panalytical, UK) at a fixed angle (173°) at 25 °C. Nanoparticle dispersions were suitably diluted before measurement.

#### Transmission Electron Microscopy (TEM)

Microscopic examination was conducted via TEM (JEM-1400; Jeol, Japan). CaP and DX@CaP dispersions were directly mounted on copper grids and dried, while Pec/DX@CaP dispersion was stained using 4% uranyl acetate before mounting and examination. Different formulations were examined for morphology and particle size analysis, where 30 measurements were recorded for image analysis (Fiji version 1.52p; National Institutes of Health, USA) (Schindelin et al. 2012).

#### Fourier Transform Infrared Spectroscopy (FTIR)

Functional group analysis was investigated using FTIR (Agilent Cary 630; Agilent Technologies, USA). Spectra of powdered samples were recorded over 4000–650 cm-1 at 2 cm-1 resolution.

### Determination of DX Loading

The amount of DX loaded was directly quantified, where the obtained DX@CaP pellet was dissolved in 1 N HCl. DX concentration in the formed solution was then determined spectrophotometrically (Agilent Cary 60; Agilent Technologies, USA) at 346 nm (El-Habashy et al. 2021). Entrapment efficiency was calculated using Eq. 1:

Entrapment efficiency (%) = (Actual drug amount in pellet)/ (Initial drug amount added) ×100%.

### In Vitro Drug Release

DX release from the developed formulations was investigated using dialysis membrane tubing (Servapor® MWCO 12,000–14,000; Serva, USA). Briefly, aliquots of DX solution (as control), DX@CaP, and Pec/DX@CaP (all corresponding to 1 mg DX) were dialyzed against 10 mL water as a release medium. Experiments were carried out at 37 °C and 50 rpm shaking (Wisebath shaking water bath; Daihan Scientific Co. Ltd, South Korea). At specific time points, samples were aliquoted for DX spectrophotometric quantification.

### Preparation of LPS

Lipopolysaccharides from Escherichia coli O111:B4 were purchased from Sigma-Aldrich Saint Louis, USA, Product Number: L2630. LPS powder was weighed carefully using digital balance, dissolved in saline, and vortexed before each injection.

### Experimental Procedures

#### Animals

The present study was conducted on 48 CD1 male mice of body weights ranging from 20 to 40 g. Mice were supplied by the animal house of the Faculty of Medicine, Alexandria University. Animals were maintained in a 12-h light/dark cycle and a 25 °C temperature with free access to food and water. The study procedures were approved by the Local Ethics Committee of Alexandria Faculty of Medicine, University of Alexandria (Ethical approval number 0305619).

#### Experimental Design

Mice were randomly divided into 6-groups, eight mice each.

- Group 1: Normal control group that received a daily saline intraperitoneal injection (IP) for 27 days.
- Group 2: Mice received blank CaP nanoparticles IP for 27 days.
- Groups 3, 4, 5, and 6: All received LPS (750 µg/kg) IP in saline for seven consecutive days for induction of neuroinflammation (Zhao et al. 2019). Then for additional 20 days, groups: 3,4,5 and 6 received daily IP injection of saline, free DX solution, DX/CaP and Pec/DX@CaP dispersed in saline, respectively. DX dose in groups 4,5 and 6 was 20 mg/kg /ml (Yadav et al. 2017; Balducci et al. 2018).

#### Behavioral Assessment

Twenty-four hours after the last drug treatment, behavioral tests were carried out to assess the cognitive behavior of the mice.

##### T-Maze Alternation Test

T-maze using spontaneous alternation test was used to evaluate working (short-term) memory. The maze was set so that all guillotine doors were raised. The animal was placed in the start area and allowed to choose a goal arm. The mouse was then confined in the chosen arm by quietly sliding the door down. After 30 s, the animal was removed. After that, the guillotine doors were raised, and the animal was replaced in the start area facing away from the goal arms. The mouse was allowed to choose between the two open-goal arms. Each trial should take no more than two minutes; one minute is the minimum possible. Ten trials were done, and the percent of correct alterations was calculated (Deacon and Rawlins 2006).

##### Novel Object Recognition Test (NOR)

NOR test evaluates short-term recognition memory. The test is based on the tendency of rodents to spend more time exploring a novel object than a familiar one. Mice were tested in an open field chamber (40 × 40 × 60 cm) with opaque walls, as described by Bevins and Besheer (Bevins and Besheer 2006). Briefly, after habituation to the open field arena, animals were allowed to explore two identical objects placed in the back left and right corners of the apparatus for 10 min in a training session. The amount of time exploring each object was video recorded. One hour later, for the test session, one of the two objects was replaced by a (novel) object. Mice were returned to the apparatus facing the opposite direction to object placement and allowed to explore the two objects for an additional 10 min. The time mice spent interacting with familiar and novel objects was video recorded. Noteworthy, Objects and arenas were thoroughly cleaned with 70% ethanol in between trials to prevent olfactory cues. Before the test, locomotor activity and visual ability were assessed among groups and no differences were found.

The preference for the novel object was calculated as the “discrimination index” using the following formula: (time with novel object − time with familiar object) / (time with novel object + time with familiar object). A positive value indicates more time investigating the novel object. A discrimination index of zero indicates equal time spent with both objects. Negative discrimination indicates poor exploration of the novel object.

After completion of the behavioral tests, mice were sacrificed under anesthesia, after 14 h of fasting. Brain tissue was extracted and dissected into two halves with hippocampus extraction. One brain half, hippocampus and cerebellum were fixed in 10% buffered formalin while the other brain half was washed with pre-cooling PBS buffer (0.01 M, pH = 7.4) then stored at − 80 °C. Heart, kidney, lung, and liver were dissected and fixed in 10% buffered formalin for histopathological examination of LPS toxicity.

#### Histopathological Examination

Fixed tissues were processed as formalin-fixed paraffin-embedded (FFPE) blocks. The blocks were cut into 5 μm sections and were stained with hematoxylin and eosin (H&E). The sections were examined to assess the histological features using different magnifications of the light microscope (Leica, Germany).

##### Interleukin-6 Immunohistochemical (IHC) Staining and Interpretation

IHC of all sections was done using the Avidin-Biotin-Peroxidase method. IL-6 primary antibody (mouse monoclonal, 10C12, Leica Biosystems) was used. Positive and negative controls were used in each run. The antibody was added to each section using the Bond-Max fully automated immunostainer (Leica Biosystems, USA). Positive control was added in each run. Negative control omitting the primary antibody was also added in each run. The IHC quantification of positive cells/ high power field (HPF) was performed on each slide using the quantitative-image analysis (Leica microsystems, Switzerland).

#### Biochemical Tests

The frozen brain tissues were homogenized in 100 mg tissue/ml cold PBS containing protease inhibitor cocktail (Sigma-Aldrich, St. Louis, MO.USA), the samples were centrifuged at 12,000 xg for 15 min and aliquots of supernatant were collected for measurement of SOD activity, TLR-4, AMPK, and Nrf2 (Zhao et al. 2019).

##### Brain Superoxide Dismutase (SOD) Activity

Colorimetric measurement of SOD was done according to the manufacturer’s instruction (Biodiagnostic, Cairo), which relied on the ability of the enzyme to inhibit phenazine methosulphate- mediated reduction of nitroblue tetrazolium dye using phosphate buffer PH 8.5, nitroblue tetrazolium, NADH, phenazine methosulphate and extraction reagent. SOD activity was normalized to tissue weight and expressed as U/g tissue (Nishikimi et al. 1972).

##### Brain TLR-4, AMPK and Nrf2

Mouse TLR-4 ELISA kit (catalog No EM0451, Fine Test, Wuhan, China), mouse AMPK ELISA kit (catalog No EM0830, Fine Test, Wuhan, China), and mouse NRF-2 ELISA kit (catalog No EM1604, Fine Test, Wuhan, China) were used to measure the brain levels of TLR-4, AMPK and NRF-2 based on sandwich enzyme-linked immune-sorbent assay technology and the samples were measured in duplicate at 450 nm in a microplate reader (Qujeq et al. 2013; Kong et al. 2015; Kabel et al. 2018).

##### Measurement of Total Protein

The supernatant of brain homogenate was used for the estimation of total protein by Lowry’s method using Folin phenol reagent with bovine serum albumin as a standard and the samples were analyzed in duplicate (Lowry et al. 1951).

The results of brain TLR-4, AMPK, and Nrf2 ELISA were normalized to total tissue proteins and expressed in ng\mg protein for TLR-4 and AMPK, and in pg\mg for Nrf2.

### Statistics

Statistical analysis was conducted using SPSS software version 25. The Shapiro-Wilk test was used to assess data normality of distribution. The data were presented as the mean ± standard deviation (SD). A one-way analysis of variance followed by an LSD test was used to test differences between groups. P values ≤ 0.05 were considered statistically significant. The correlation between AMPK and other measured parameters was assessed using Pearson correlation.

## Results

### Preparation of Novel Pec/DX@CaP

**Fig. 1:**
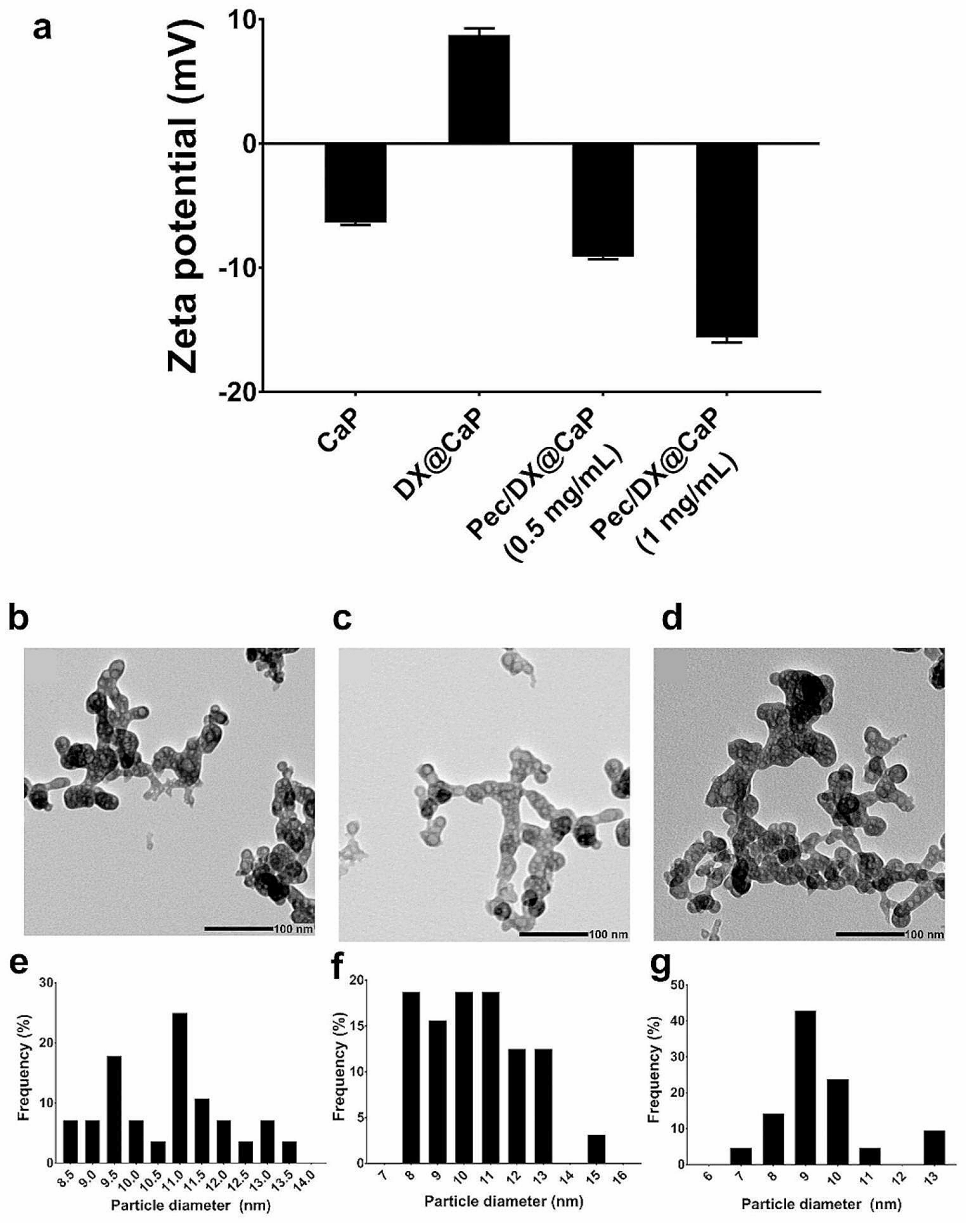
Zeta potential measurement of the developed formulations, *n* = 3 **(a)**. TEM micrographs and histograms of particle diameter for CaP **(b, e)**, DX@CaP **(c, f)**, and Pec/DX@CaP **(d, g).** Scale bar: 100 nm, *n* = 30

Zeta potential was investigated for CaP, DX@CaP, and Pec/DX@CaP, using two concentrations for pectin solution (0.5 and 1 mg/mL). Results of zeta potential measurements (Fig. 1a) show that CaP originally possessed an overall negative charge (-6.34 ± 0.2), which was reversed for DX@CaP (8.78 ± 0.5), confirming DX loading onto CaP. Coating DX@CaP with pectin resulted in charge reversal for both tested concentrations, which was higher for 1 mg/mL (15.6 ± -0.4), further verifying successful pectin coating. These findings were similar to previous data (Ni and Fox 2019), where the surface of the pectin/hydroxyapatite hybrid presented more surface negativity than plain hydroxyapatite, illustrating a strong electrostatic interaction.

Since charge reversal and pectin coating were successfully achieved using 0.5 mg/mL pectin solution, the corresponding pectin-coated DX@CaP dispersion was selected for further studies and referred to as Pec/DX@CaP.

### TEM

TEM was conducted for the investigation of particle size analysis and morphological examination of CaP, DX@CaP, and Pec/DX@CaP.

As seen in Fig. 1b–d, all the prepared particles possessed a spherical, round morphology. Particle size distribution revealed a nano-size range, where CaP, DX@CaP, and Pec/DX@CaP possessed average particle diameters of 10.7 ± 1.3 nm, 10.47 ± 1.8 nm, and 9.44 ± 1.47 nm, respectively (Fig. 1e–g). These results agree with previous data (Ni and Fox 2019), where similarly prepared calcium phosphate nanoparticles were 10–25 nm in diameter.

### FTIR

**Fig. 2:**
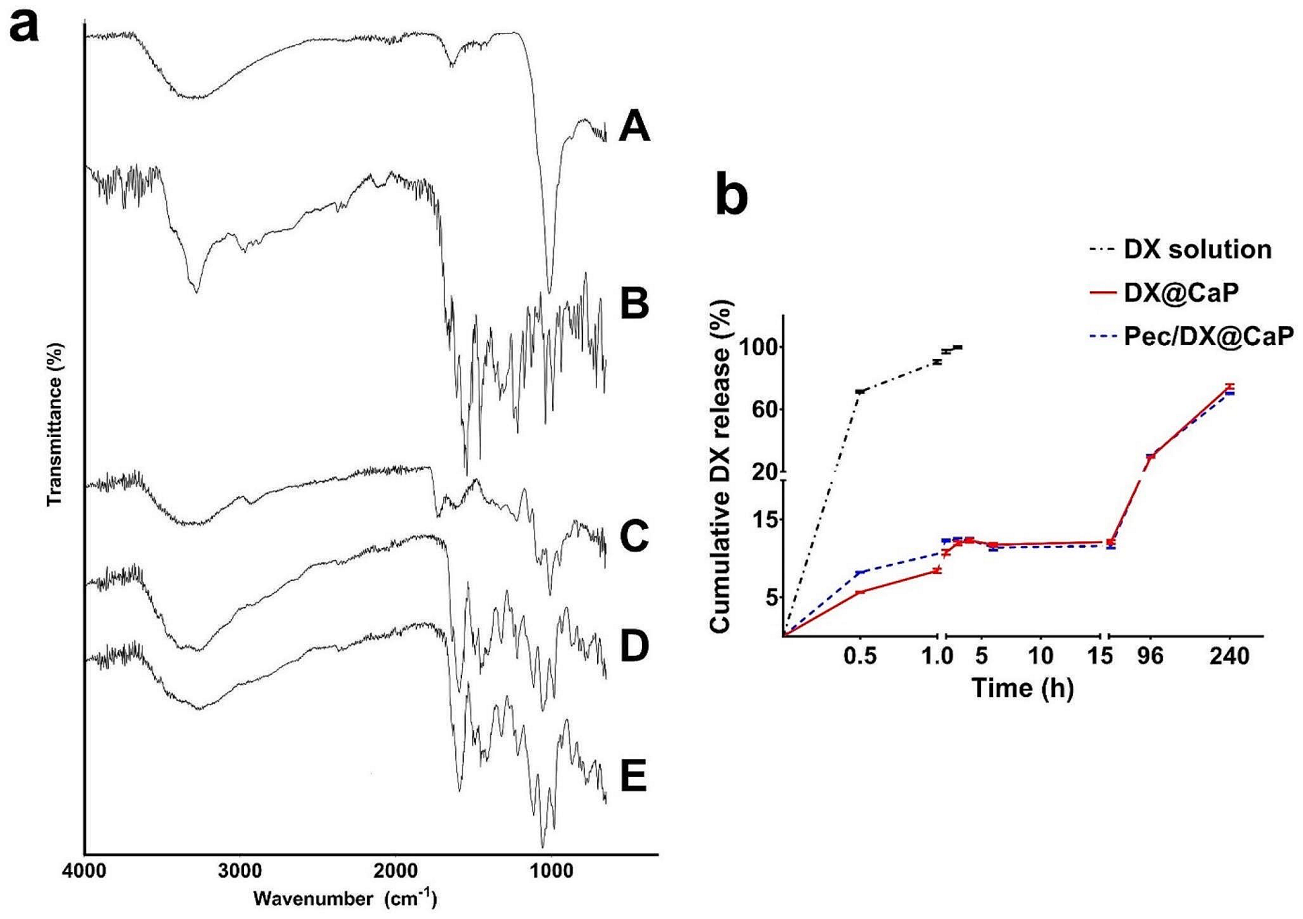
Fourier transform infrared spectra **(a)** for CaP (A), DX (B), pectin (C), DX@CaP (D), and Pec/DX@CaP (E). In vitro cumulative DX release from different formulations **(b)**, showing controlled release from both DX@CaP and Pec/DX@CaP compared to DX solution, *n* = 3

FTIR analysis was conducted for CaP, DX@CaP, and Pec/DX@CaP in comparison to raw DX and pectin (Fig. 2a). The spectrum for CaP shows a stretching band at 3550 cm-1, corresponding to hydroxyl groups, while the broad peak at 3300 cm-1 corresponds to adsorbed water. On the other hand, phosphate stretching is indicated by the band at 1010 cm-1 (El-Habashy et al. 2021). DX spectrum shows typical bands for C-H stretching, O-H/N-H stretching and N-H bending at 2880 cm-1, 3300 cm-1, and 1570 cm-1, respectively (El-Habashy et al. 2021). The pectin spectrum shows peaks characteristic of carbohydrates at 3350 cm-1 (O-H stretching), 1735 cm-1 (C = O stretching), 1605 cm-1 (COO- band), and 1010 cm-1 (COO-). As can be seen (Fig. 2A), both spectra for DX@CaP and Pec/DX@CaP show peaks of individual components. The slight change in peak position and intensity (carboxylate at 1010 cm-1) for Pec/DX@CaP can be attributed to the electrostatic interaction between pectin as a coating polymer for DX@CaP (Chang et al. 2017).

### Determination of DX Loading

Quantification of DX content in the prepared DX@CaP was directly evaluated. DX@CaP exhibited an entrapment efficiency of 48.34 ± 2.9% and drug loading values reached 0.8 ± 0.05 mg DX/mg CaP. These results are in accordance with our zeta measurements and further confirm successful DX loading.

### In Vitro Drug Release

Drug release from delivery systems is an essential parameter that influences drug pharmacologic behavior in the biological system. In our study, DX release from DX@CaP and Pec/DX@CaP was investigated in comparison to the DX solution.

Release profiles (Fig. 2b) reveal that DX was completely released from the dialysis bag for DX solution, reaching 71.2 ± 0.6% in 0.5 h and 99.6 ± 0.5% after 3 h. This result confirms the efficient dialyzability of DX in the applied experimental setting. On the other hand, a significantly (*p* ≤ 0.05) lower release rate was detected for both nanoparticle formulations. A relatively biphasic profile was obtained, where both DX@CaP and Pec/DX@CaP achieved only 12.1 ± 0.2% and 11.6 ± 0.2% DX release after 24 h, respectively. Afterward, a higher-release rate was detected, where both formulations could achieve 74.6 ± 1.4% and 70.2 ± 0.4% after 10 days. Such a controlled release pattern is generally beneficial, especially for the amelioration of DX dose-dependent cytotoxic actions (Harland et al. 2020).

### Behavioral Assessment

**Fig. 3:**
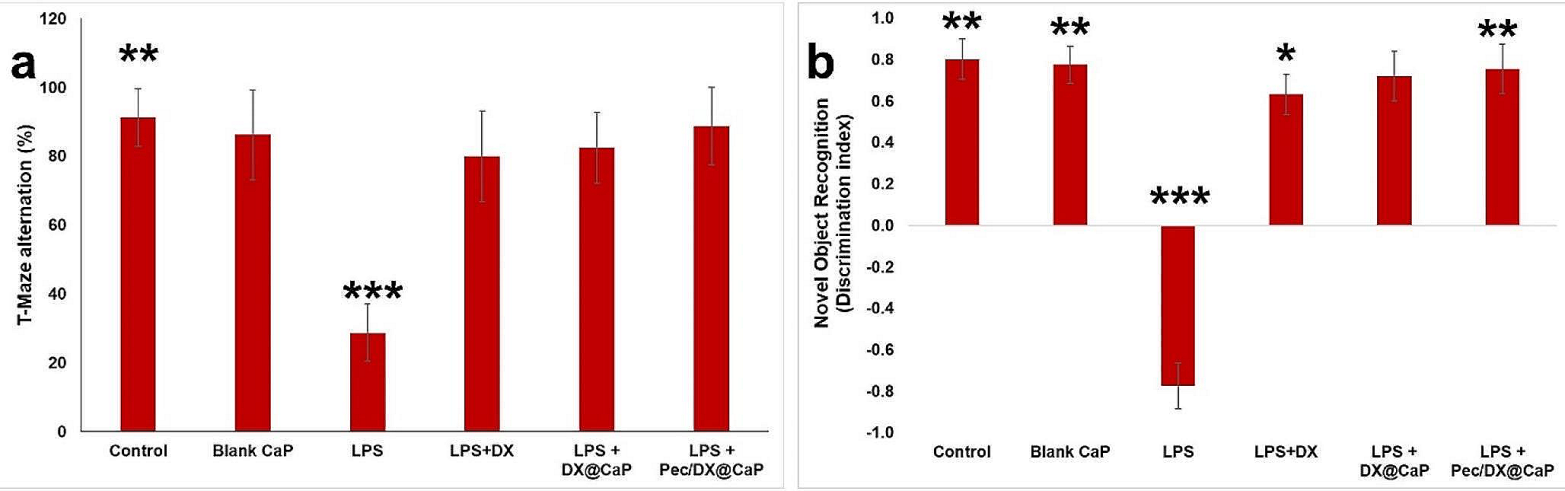
Effect of treatment of free DX, DX@CaP, and Pec/DX@CaP, on percentage T- maze alternation **(a)** and discrimination index **(b)**, in comparison to normal control group, blank CaP nanoparticle treated group and LPS (untreated) group. *n* = 8. (*: Significant difference in comparison to LPS (untreated) groups, **: Significant difference in comparison to LPS (untreated) and free DX groups, ***: Significant difference in comparison to all other groups)

Doxycycline, DX@CaP, and Pec/DX@CaP improved LPS-induced impairment of working memory and short-term recognition, tested by T- maze spontaneous alternation tests and NOR respectively (Fig. 3a and b).

T- maze spontaneous alternation test was conducted to assess the working memory. It showed statistically significant reduction of the percent of correct alternations among LPS (untreated) mice in comparison to the normal control group. Doxycycline, DX@CaP, and Pec/DX@CaP- treatment achieved significant improvement in the percent of correct alternations denoting improvement of the working memory.

NOR test revealed that LPS (untreated) animals had negative discrimination indices, indicating poor exploration of novel objects. Doxycycline treatment resulted in significant improvement in comparison to the LPS (untreated) group, however, a significant difference was still present between this group and the normal control group. On the other side, the DX@CaP and Pec/DX@CaP resulted in more improvement that makes insignificant differences between them and the normal control group.

### Histopathological Examination

**Fig. 4:**
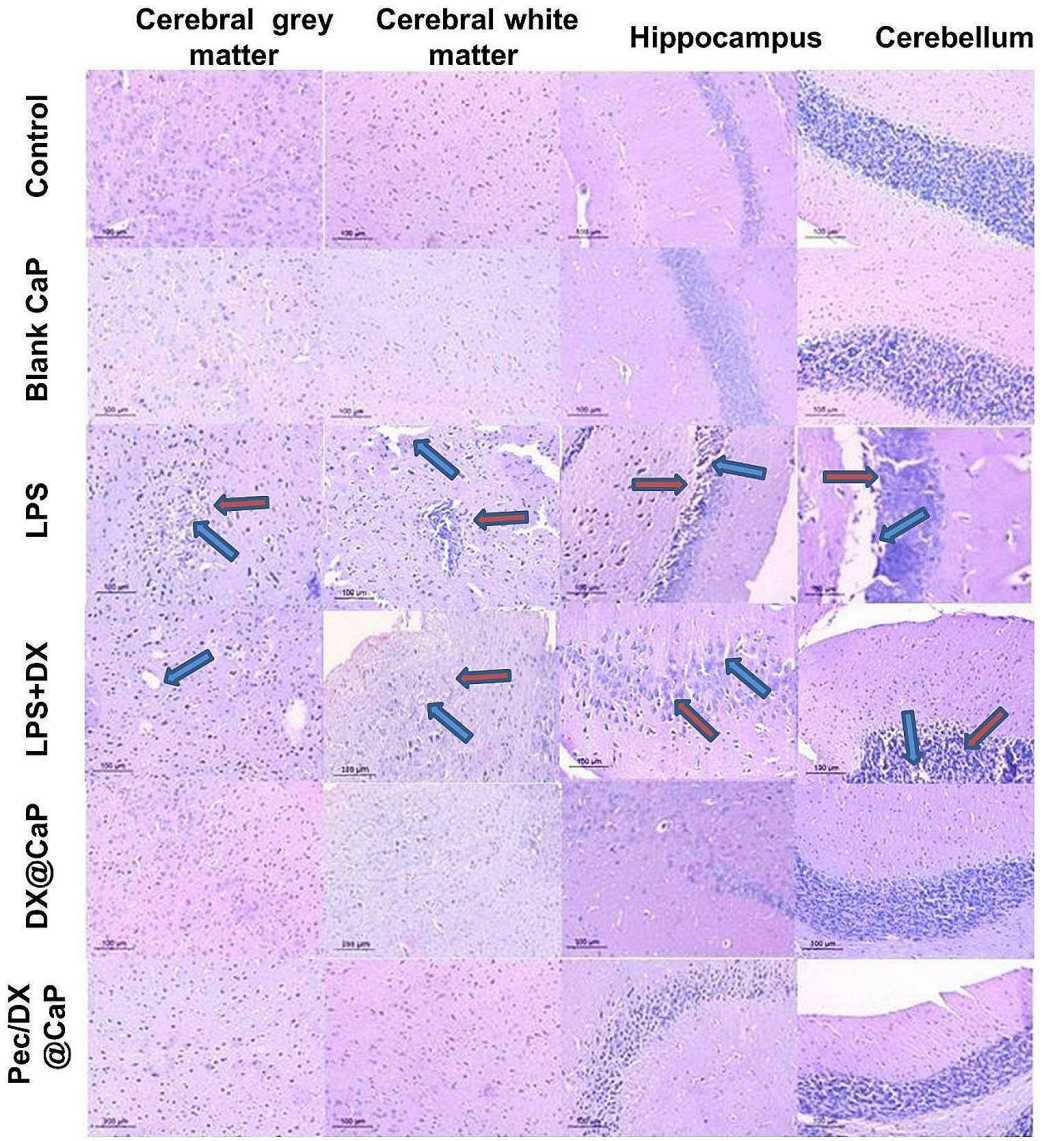
Microscopic examination of H& E-stained tissues of cerebral grey, white matter, hippocampus, and cerebellum showing a normal histological architecture of the cerebrum, hippocampus, and cerebellum in the normal control and blank CaP groups. In the LPS (untreated) group, the cerebral grey and white matter, the hippocampus, and the cerebellum showed inflammatory infiltrate, cellular degeneration (red arrow), and oedema (blue arrow). These features were mildly decreased in the LPS/DX group, while the DX@CaP group demonstrated a marked decrease in the inflammatory infiltrate and oedema. The Pec/DX@CaP group revealed normal architecture similar to the normal control group (H&E, x200). *n* = 8

Examination of H&E-stained brain tissues using microscopic examination showed normal histological architecture of the cerebrum, hippocampus, and cerebellum in the normal control and blank nanoparticles-treated groups (Group 1 and 2). On the other hand, in the LPS (untreated) group (Group 3), the cerebral grey and white matter, the hippocampus, and the cerebellum showed inflammatory infiltrate, cellular degeneration, and oedema. These features were mildly decreased in the doxycycline-treated group (Group-4), while the DX@CaP-treated group (Group-5) demonstrated a marked decrease in the inflammatory infiltrate and oedema. The Pec/DX@CaP-treated group (Group-6) revealed normal architecture like the normal control group. (Fig. 4). LPS toxicity histopathological results are available in (supplementary Fig. 1).

#### Brain IL6 Level

**Fig. 5:**
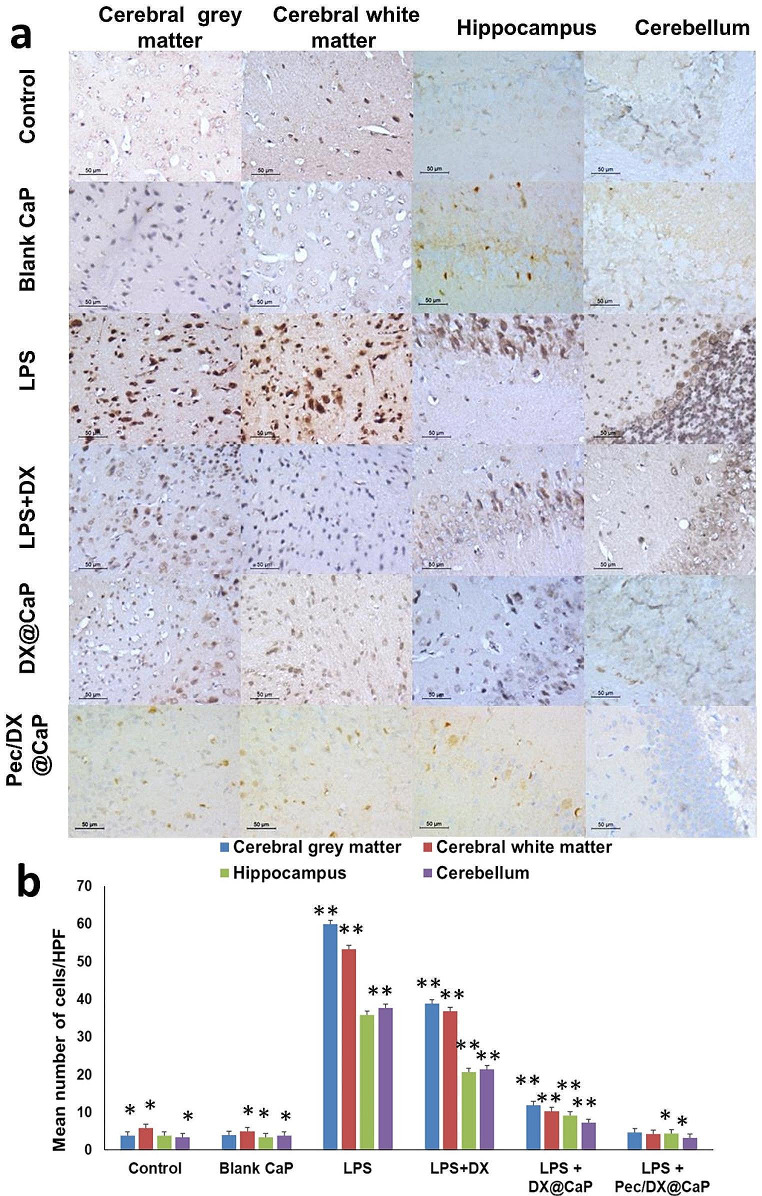
Assessment of the level of IL-6-stained cells in the cerebrum, hippocampus, and cerebellum using Immunohistochemistry. *n* = 8. (Immunohistochemistry, x400) **(a).** The effect of treatment of free DX, DX@CaP, and Pec/DX@CaP, on percentage IL-6 expression **(b)**, assessed by immunohistochemistry, in comparison to control untreated group, blank CaP nanoparticle and LPS(untreated) group in cerebral grey matter, cerebral white matter, hippocampus and cerebellum. *n* = 8 (*: Significant difference in comparison to LPS (untreated), free DX and DX@CaP groups **: Significant difference in comparison to all other groups)

The level of IL-6-stained cells in the cerebrum, hippocampus, and cerebellum was assessed using immunohistochemistry (Fig. 5a and b). The normal control and blank CaP-treated groups (Group 1 and 2, respectively) showed normal levels of IL-6 positively stained cells (means ± SD for Group 1 were 3.8 ± 0.6, 5.8 ± 0.8, 3.8 ± 0.6, and 3.4 ± 0.6 in cerebral grey matter, cerebral white matter, hippocampus, and cerebellum, respectively). For Group 2 (means ± SD of IL-6 positively stained cells numbers were 3.9 ± 0.5, 4.9 ± 0.4, 3.4 ± 0.7, and 3.8 ± 0.6 in cerebral grey matter, cerebral white matter, hippocampus, and cerebellum, respectively). Group 3 (LPS-(untreated) group) showed a significant increase in the levels of IL-6 - positively stained cells in all studied areas (means ± SD of IL-6 positive cells numbers were 61.8 ± 2.7, 53.3 ± 1.7, 35.8 ± 2.2, and 37.6 ± 2.1 in cerebral grey matter, cerebral white matter, hippocampus, and cerebellum, respectively).

The mean number of IL-6 positively stained cells decreased in the free doxycycline-treated (Group-4) to be (38.9 ± 2.0, 36.9 ± 2.1, 20.6 ± 1.3, and 21.4 ± 1.1) in cerebral grey matter, cerebral white matter, hippocampus, and cerebellum, respectively). In Group 5 (DX@CaP -treated group), more significant improvement was identified, where the means ± SD for IL-6 positively stained cells were (11.9 ± 1.1, 10.2 ± 0.5, 9.1 ± 0.6, and 7.2 ± 0.7) in cerebral grey matter, cerebral white matter, hippocampus, and cerebellum, respectively. Pec/DX@CaP -treated group (Group-6), showed maximal improvement where the mean value of IL-6 decreased significantly (4.6 ± 0.4, 4.3 ± 0.4, 4.4 ± 0.9, and 3.2 ± 0.2 in the cerebral grey matter, cerebral white matter, hippocampus, and cerebellum, respectively, with an insignificant difference in comparison to Group 1 and 2.

### Brain SOD, TLR-4, AMPK and Nrf2

**Fig. 6:**
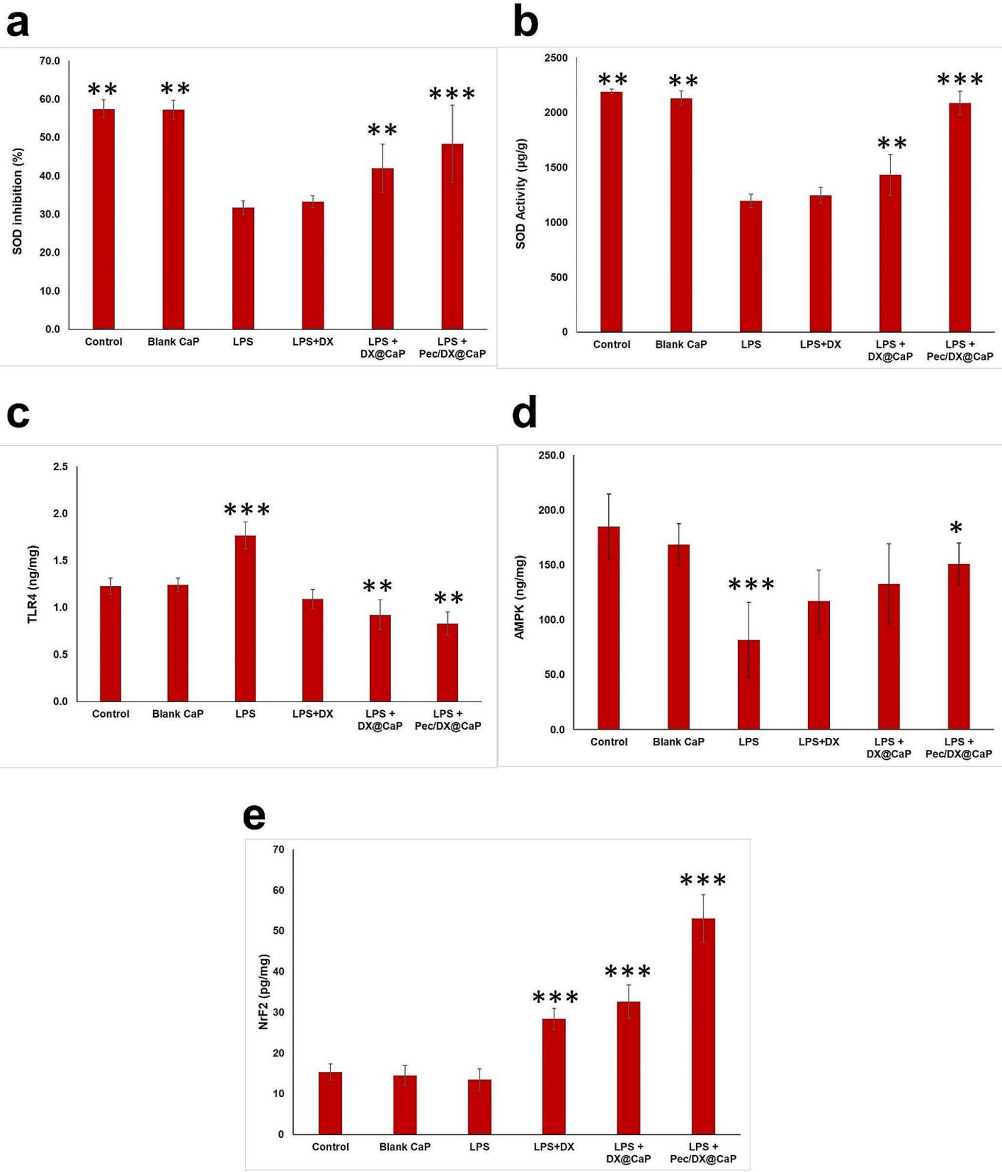
Effect of treatment of free DX, DX@CaP, and Pec/DX@CaP, on percentage superoxide dismutase activity inhibition **(a)**, superoxide dismutase activity (µg/g) **(b)**, the expression of toll-like receptor-4 (TLR4; ng/mg) **(c)**, adenosine monophosphate kinase (AMPK; ng/mg) **(d)** and nuclear factor erythroid 2-related factor 2 (Nrf2; pg/mg) **(e)** in brain tissue, in comparison to normal control, blank CaP treated and LPS (untreated) group. *n* = 8. (*: Significant difference in comparison to LPS (untreated), **: Significant difference in comparison to LPS (untreated) and free DX groups, ***: Significant difference in comparison to all other groups)

Lipopolysaccharide administration resulted in the depletion of the antioxidant SOD in the brain tissue homogenate, denoting the occurrence of oxidative stress. Doxycycline treatment could not produce a significant improvement in the SOD activity in comparison to the LPS (untreated) group, however, the two doxycycline nanoparticle preparations resulted in a significant increase in the SOD activity. Improvement of the antioxidant activity was more prominent with Pec/DX@CaP in comparison to DX@CaP and free doxycycline. (Fig. 6a and b)

The cellular expression of TLR-4 in the studied brain tissue was elevated in LPS (untreated) mice. Doxycycline treatment reduced the TLR-4 expression, resulting in an insignificant difference between the free DX-treated and the normal control groups. Both DX@CaP and Pec/DX@CaP reduced TLR4 to a lower level compared to the normal control group. (Fig. 6c)

Mice receiving LPS showed a significant decrease in the level of AMPK in the brain tissue homogenate in comparison to the normal control group, an effect that was antagonized by doxycycline treatment. AMPK-upregulation was notably higher among Pec/DX@CaP treated mice. (Fig. 6d)

Nrf2 levels were similar for LPS (untreated) group and normal control group. Doxycycline treatment induced a more prominent increase in Nrf2 in comparison to both normal control and LPS (untreated) mice. The maximal increase was recorded among Pec/DX@CaP treated group. (Fig. 6e)

### Correlation Studies

**Fig. 7:**
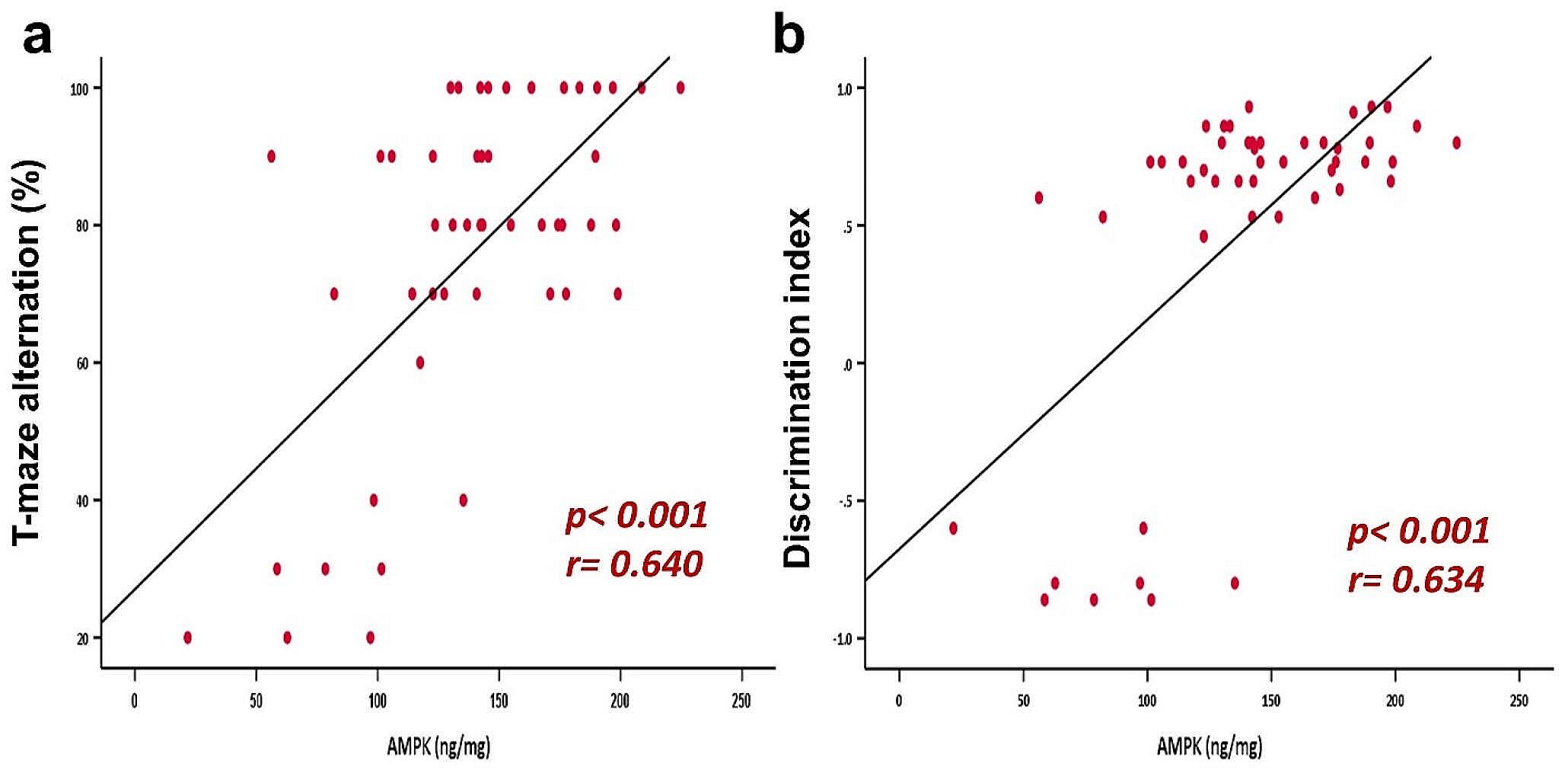
Correlation between AMPK (ng/mL) and T-maze alternation percentage **(a)**, and discrimination index in the novel recognition test **(b)**

**Fig. 8:**
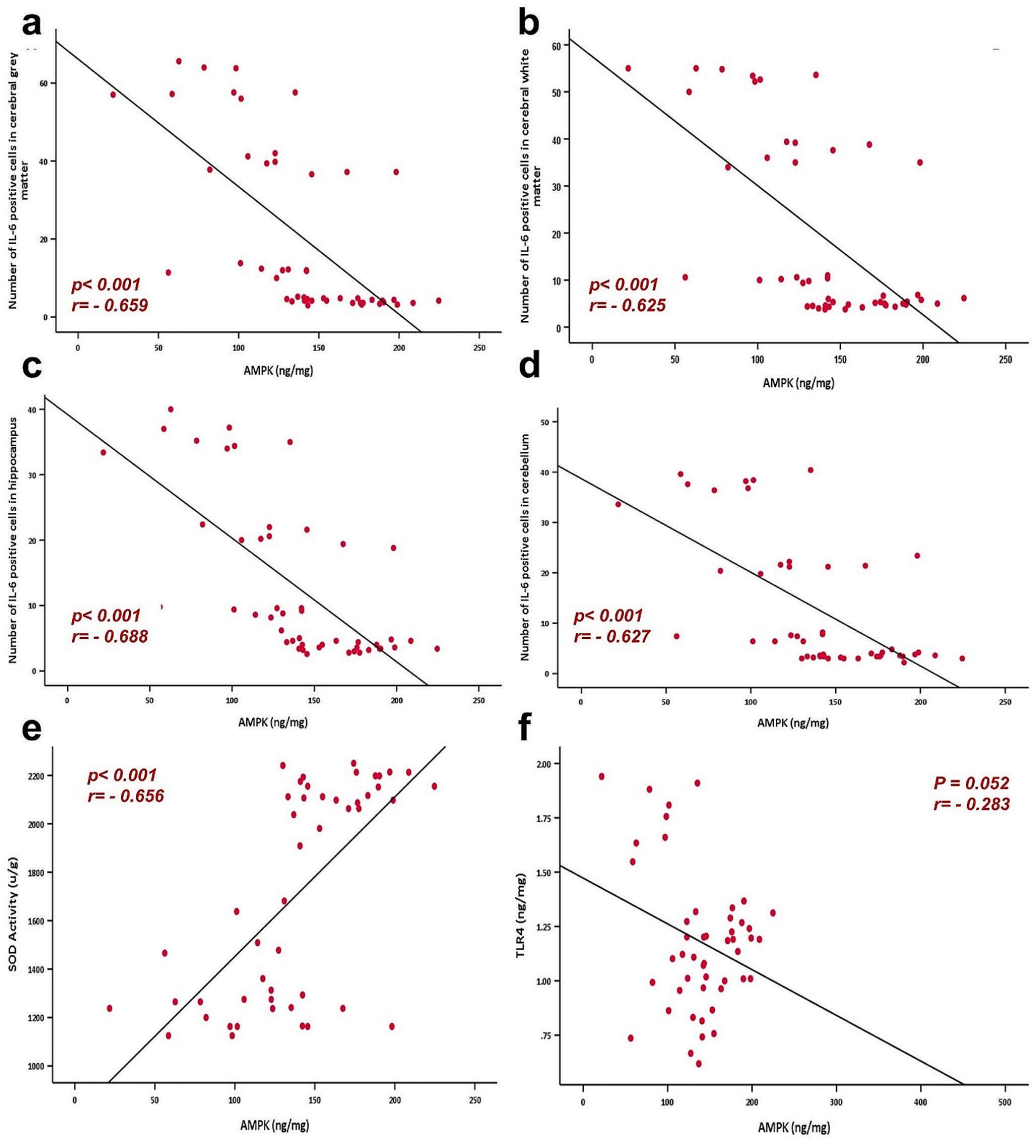
Correlation between AMPK (ng/mL) and number of IL-6 positive cells in cerebral grey matter **(a)**, cerebral white matter **(b)**, hippocampus **(c)**, cerebellum **(d)**. Correlation between AMPK (ng/mL) and SOD activity (µg/g) **(e)** and TLR-4 expression (ng/mg) **(f)**

Testing the correlation between AMPK and other tested parameters showed a negative correlation with the number of IL-6 positive cells in cerebral grey and white matter, hippocampus, and cerebellum, in addition to TLR-4. While there was a positive correlation between the T-maze alternation percentage and the discrimination index in novel recognition test (Figs. 7 and 8).

## Discussion

Neuroinflammation is a major pathologic feature of numerous neuropsychiatric diseases. It starts in response to multiple triggers to limit their injurious effects. However, failure to stop the inflammation by the endogenous anti-inflammatory pathways induces uncontrolled synthesis and release of inflammatory mediators, tissue damage, ending with permanent neuronal damage (Harland et al. 2020).

The current study was conducted to assess the potential effect of DX and different DX-loaded nanoparticle preparations; DX@CaP and Pec/DX@CaP, on LPS-induced neuroinflammation in mice, highlighting the role of TLR-4, AMPK, and Nrf2. Also, the impact of neuroinflammation on histopathological features and behavioral changes was elaborated.

LPS is a component of the cell membrane of Gram-negative bacteria (Pulido-Salgado et al. 2018), reportedly related to various neurodegenerative diseases. Most commonly, there is an increase in LPS physiological concentrations during infection. In addition, LPS from intestinal leaks shows higher levels during stress or as a result of high-fat diets (Moreira et al. 2012; Gárate et al. 2013). Unfortunately, high LPS serum levels are closely associated with the development of Parkinson’s disease, Alzheimer’s disease, and other neurological disorders. Peripheral LPS injection is associated with an increase in cytokine production in the CNS. It has been approved as a model of induced neuroinflammation, in a trial to identify new therapeutic strategies for the treatment of neurodegenerative diseases (Szandruk-Bender et al. 2022). LPS mediates its actions via multiple receptors, among which, TLR-4 in the CNS forms a complex with LPS which results in the activation of transcription factors and enhanced expression of cytokines including IL-6 (Park et al. 2009).

Our results indicated that the IP injection of LPS induced a significant decrease in the levels of SOD and AMPK in the brain tissue, in addition to the upregulation of TLR-4, confirming the establishment of neuroinflammation. Successful LPS brain access can be justified firstly by its passage through CNS structures lacking the BBB for instance the meninges, choroid plexus, and circumventricular organs. These areas have been proposed as neuroinflammatory sensor regions (Qin et al. 2007; Morita et al. 2016). The second explanation of the central effects of the peripheral LPS is its destructive effect on the BBB (Peng et al. 2021). Indeed, excessive TLR signaling, and IL-6 expression have been linked to several inflammatory diseases (Yan 2006). Cells with activated TLR-4 show translocation of high mobility group box 1 (HMGB1) nuclear protein to the cytoplasm, then to the extracellular space, where it serves as a “danger signal” with subsequent cytokine release (Tadie et al. 2012). In the present study, these excessive and inappropriate inflammatory reactions were evident in LPS (untreated) mice brain and were accompanied by prominent changes in the histological features of the cerebral white and grey matter, hippocampus, and cerebellum, where inflammatory infiltrate, cellular degeneration, and oedema were identified. The levels of IL-6 were significantly increased in all studied brain areas. Additionally, the significant memory impairment recorded by NOR and T-maze tests, was considered as a proof of cognitive dysfunction. In addition, LPS (untreated) mice had decreased AMPK expression. AMPK is an enzyme responsible for sensing the energy status of the cell. It is crucial for adaptable reactions in different physiological and pathological circumstances. It is activated by stresses that result in increased cellular AMP concentration compared to ATP (e.g., glucose deprivation or hypoxia) or a rise in energy expenditure. AMPK is active in the phosphorylated form, where it exerts anti-inflammatory and antioxidant actions counteracting the damaging effect of an inflammatory state (Dasgupta and Chhipa 2016). Previous studies showed that LPS can stimulate the dephosphorylation of AMPK, which results in suppression of the inhibitory activity of AMPK on the mammalian target of rapamycin (mTOR). In addition to repression of autophagy via uncoordinated-51-like kinase 1 (ULK1), which may increase the severity of LPS-induced inflammatory injury (Fan et al. 2018). This inflammatory cascade overactivity results in ROS overproduction, which induces lipid peroxidation, leading to free radical release. That is why LPS-induced suppression of AMPK can be involved in the prominent neuroinflammatory changes and increase IL-6 expression recognized in LPS (untreated) mice, with subsequent deficits in both learning and memory (Li et al. 2020). As a counter-regulatory mechanism, antioxidants like SOD start to catalyze active oxygen intermediates formation, which scavenges free radicals, to suppress the process of peroxidation. Continued inflammation and oxidative stress end with suppression of the SOD activity and failure of the cell to halt the tissue damage (Han et al. 2019). As such, it is implied that pharmacological restoration of AMPK activation may be helpful in the treatment of inflammatory disorders.

Nrf2 is a transcription factor with an anti-inflammatory effect. It is activated in the microglia in response to LPS exposure to start negative feedback suppression of the neuroinflammatory reaction. However, in the current study, the modest elevation of Nrf2 was unable to counteract the LPS-induced changes (Bao et al. 2021).

Doxycycline, the well-known synthetic tetracycline used since 1967 as an antibacterial agent is now seen from a different perspective. Recent research studied its anti-inflammatory, antioxidant, anti-amyloidogenic, and anti-apoptotic activities (Altoé et al. 2021). The anti-inflammatory and antioxidant effects of doxycycline on the skin and the impact of these activities on wound healing can increase the level of antioxidant enzymes, namely, SOD and catalase, and improve wound healing. They attributed these effects to the suppression of the activity of cyclooxygenase-2 (COX-2) and so the production of prostaglandin E2. Other studies showed the promising activity of doxycycline against traumatic brain injury. It was found that doxycycline can improve memory and locomotor activity, reduce neuroinflammation, in addition to increasing the antioxidant enzyme activities and the neurotransmitter level (Rana et al. 2020).

Nanodrug delivery is an efficient approach for modification of drugs therapeutic efficacy. In this study, we adopted simple chemical precipitation for the preparation of CaP. Wet chemical precipitation is a very popular technique for the fabrication of various types of calcium phosphate nanoparticles. Changing processing parameters allows for the production of nanoparticles with variable physical and functional features. We further employed simple surface adsorption to prepare DX@CaP, where DX loading occurs mainly due to Van Der Waals forces without affecting DX chemical integrity. Finally, the developed DX@CaP was coated with pectin by electrostatic deposition via a simple titration technique. TEM examination confirmed that DX loading and further pectin coating resulted in no pronounced change in particle size. In addition, in vitro release studies indicated the convenience of the developed nanoparticles for the prospective pharmacologic functionality (Schindelin et al. 2012; Shehata et al. 2022).

Pec/DX@CaP showed a significant improvement in cognitive function as evidenced by memory testing using T-maze and NOR tests and reduction of the histopathological manifestations of neuroinflammation identified by microscopical examination of H&E-stained sections of multiple brain areas. Pec/DX@CaP could reverse the histological inflammatory findings identified in the LPS-induced neuroinflammation, resulting in near normal histological picture of the cerebral white and grey matter, the hippocampus, and the cerebellum, in addition to normalization of the level of IL-6 positively stained cells in these areas. Also, Pec/DX@CaP treatment was associated with an increase in the antioxidant SOD and Nrf2. Moreover, Pec/DX@CaP treatment induced a significant increase in the level of AMPK and suppression of the expression of TLR-4. A negative correlation was identified between AMPK and TLR-4, denoting the immunomodulatory effect of doxycycline that was significantly enhanced by nanoparticle loading. The immunomodulatory effect of doxycycline was recognized by a recent study that concluded a doxycycline attenuating effect on TLR-induced immunological reaction against bacterial infection. This makes it a dual-edged sword, despite its risk to suppress immunity, in case of misdescription of this antimicrobial, it has a great possibility for protection against diseases with uncontrolled inflammation (Silva Lagos et al. 2022). The immunomodulatory effect of doxycycline can be explained by several suggested mechanisms. Firstly, its strong chelating properties, secondly, its ability to inhibit protein kinase C (PKC), with subsequent suppression of the LPS-stimulated cytokine secretion like IL-1 and IL-6 (Bode et al. 2014). Thirdly, its inhibitory action on the small mitochondrial ribosome, with inhibition of mitochondrial translation (Fuentes-Retamal et al. 2020). Lastly, doxycycline ability to suppress important sepsis-related pro-inflammatory cytokines, mainly the NF-κB signaling pathway (Patel et al. 2020).

Assessment of the correlation between AMPK and the other studied parameters has elucidated that AMPK was positively correlated with all signs of neuroinflammation improvement, namely the histopathological features of inflammation, the IL-6 expression in the brain tissues, and the level of the antioxidant SOD in addition to the cognitive function. On the other side, it was negatively correlated with TLR-4 expression. Surprisingly, there was a non-significant correlation between AMPK and Nrf2. However, the current study showed positive results regarding Nrf2 brain levels reflecting its anti-inflammatory role, this finding can be explained by the fact that the intracellular level of Nrf2 is regulated by the equilibrium between its synthesis and degradation. Under normal physiological conditions, the Nrf2 has a half-life of 10–30 min. Its level is kept low by the effect of Keap1-mediated degradation. However, in the case of oxidative stress, Keap1 undergoes oxidations and structural changes preventing the degradation of Nrf2, which spreads into the nucleus to control the expression of numerous genes encoding the synthesis for proteins essential to maintaining cellular homeostasis (Guerrero-Hue et al. 2020; Lin and Yao 2020). The highest Nrf2 level was identified among the Pec/DX@CaP treated group, which was associated with the maximal improvement in histological, cognitive, and biochemical parameters. Previous studies have demonstrated that various inflammatory and oxidative stress-associated disorders can be significantly improved by enhancement of the level of Nrf2 (Lv et al. 2017; Guerrero-Hue et al. 2020).

## Conclusion

The current study showed a significant anti-neuroinflammatory effect of DX and highlighted the added value of the promising nanocarrier Pec/DX@CaP in reversing LPS-induced neuroinflammation with subsequent cognitive behavior improvement. The underlying mechanisms of such favorable effects involve enhancing AMPK, which in turn is associated with the suppression of TLR-4 and enhancement of Nrf2 expression.

## Electronic Supplementary Material

Below is the link to the electronic supplementary material.

Supplementary Material 1

## Data Availability

Data and material will be available upon request.

## Abbreviations

DX: Doxycycline
DX@CaP: Doxycycline loaded calcium phosphate
Pec/DX@CaP: Pectin coated doxycycline loaded calcium phosphate nanoparticles
LPS: Lipopolysaccharide
AMPK: Adenosine monophosphate activated protein kinase
