# Supplementary material for: Doxycycline-Loaded Calcium Phosphate Nanoparticles with a Pectin Coat Can Ameliorate Lipopolysaccharide-Induced Neuroinflammation Via Enhancing AMPK
Source: J Neuroimmune Pharmacol. 2024 Jan 18;19(1):2. doi: 10.1007/s11481-024-10099-w (PMC10796490; doi:10.1007/s11481-024-10099-w)
Supplement: Supplementary file 1 — Supplementary Material 1 [file 11481_2024_10099_MOESM1_ESM.pdf]

Title: Doxycycline-Loaded Calcium Phosphate Nanoparticles with a Pectin Coat Can Ameliorate Lipopolysaccharide-Induced Neuroinflammation Via Enhancing AMPK

Authors:

Suzan Awad AbdelGhany Morsy [1], Mona Hassan Fathelbab [2], Norhan S. El-Sayed [3], Salma E. El-Habashy [4], Rania G Aly [5], Sahar A. Harby [6].

Affiliations:

1. Clinical Pharmacology Department, Faculty of Medicine, Alexandria University,Alexandria, Egypt.
   SUSAN.ULGHANI@alexmed.edu.eg
   https://orcid.org/0000-0002-5874-194X
2. Medical Biochemistry Department. Faculty of Medicine, Alexandria University, Alexandria, Egypt.
   mona.fathelbab@alexmed.edu.eg
   https://orcid.org/0000-0002-2752-7609
3. Medical Physiology Department. Faculty of Medicine, Alexandria University, Alexandria, Egypt.
   Norhan.mohamed@alexmed.edu.eg
   https://orcid.org/0000-0003-4270-8544
4. Department of Pharmaceutics. Faculty of Pharmacy, Alexandria University, Alexandria, Egypt.
   dr.salma.essam@hotmail.com
   https://orcid.org/0000-0003-3891-5049
5. Pathology Department. Faculty of Medicine, Alexandria University, Alexandria, Egypt.
   rgm2006isa@yahoo.com
   https://orcid.org/0000-0003-1227-6357
6. Clinical Pharmacology Department, Faculty of Medicine, Alexandria University, Alexandria, Egypt.
   sahar.ahmed15@alexmed.edu.eg
   https://orcid.org/0000-0002-3782-7541

Corresponding authors:

Sahar A. Harby

Clinical Pharmacology Department. Faculty of Medicine, Alexandria University, Alexandria, Egypt

E-mail address: sahar.ahmed15@alexmed.edu.eg

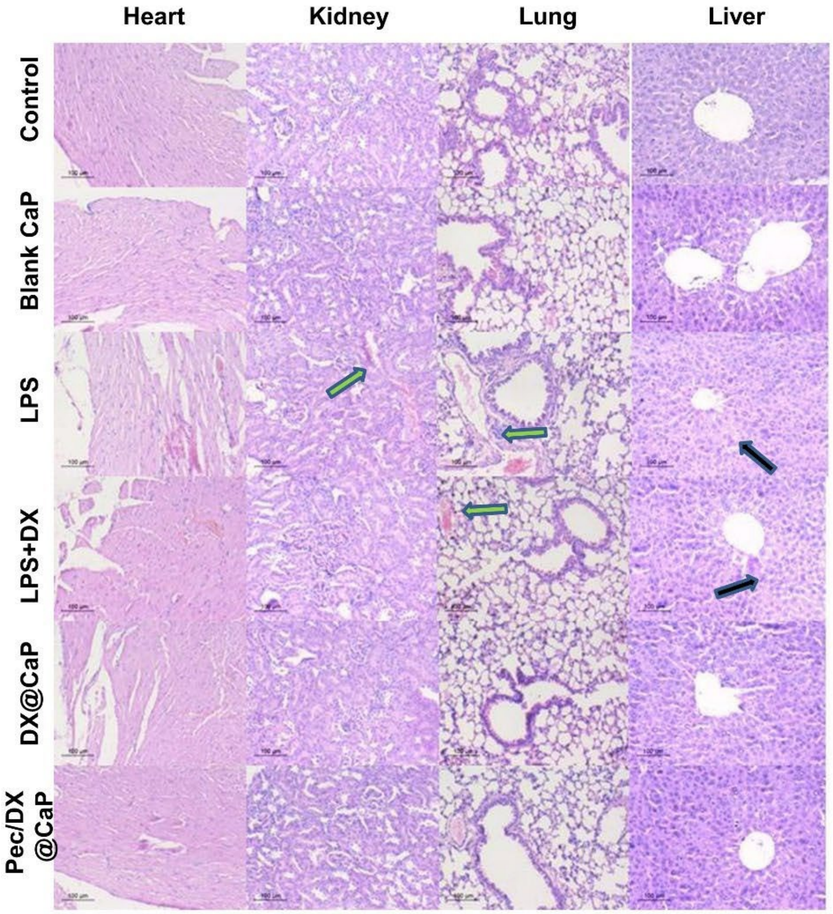

Supplementary Figure 1: LPS toxicity study

- The dissected organs (heart, kidney, lung, and liver) show normal architecture in all groups except for vascular congestion (green arrow) in LPS (untreated) and DX-treated groups as well as hepatocyte ballooning and microsteatosis (black arrow) which decreases remarkably in the DX@CaP, and Pec/DX@CaP-treated groups • Compared to the normal group and the group treated by blank nanoparticles which have a normal histological architecture of the cerebrum, hippocampus, and cerebellum, in the LPS (untreated) group, the cerebrum (grey and white matters).
